# *Chlamydomonas* FAP265 is a tubulin polymerization promoting protein, essential for flagellar reassembly and hatching of daughter cells from the sporangium

**DOI:** 10.1371/journal.pone.0185108

**Published:** 2017-09-20

**Authors:** Damayanti Tammana, Trinadh Venkata Satish Tammana

**Affiliations:** Institute of Bioinformatics and Applied Biotechnology (IBAB), Bangalore, Karnataka, India; Consiglio Nazionale delle Ricerche, ITALY

## Abstract

Tubulin polymerization promoting proteins (TPPPs) belong to a family of neomorphic moon lighting proteins, involved in various physiological and pathological conditions. In physiological conditions, TPPPs play an important role in microtubule dynamics regulating mitotic spindle assembly and in turn cell proliferation. In pathological situations, TPPPs interact with α-synuclein and β-amyloid and promote their aggregation leading to Parkinson’s disease and multiple system atrophy. Orthologs of TPPP family proteins were identified in ciliary proteomes from various organisms including *Chlamydomonas* but their role in ciliogenesis was not known. Here we showed that Flagellar Associated Protein, FAP265, a *Chlamydomonas* homologue of TPPP family proteins, localizes in the cytosol, at the basal bodies and in the flagella of vegetative *Chlamydomonas* cells. During cell division, the protein was found as a distinct spot in the nucleus and at the cleavage furrow which forms between the daughter cells. Further null mutants of *Chlamydomonas* FAP265 protein, *fap265*, showed severe defects in hatching from the mother sporangium. Daughter cells of *fap265* were significantly larger in size compared with wild type cells. Moreover, the daughter cells present within the mother sporangium failed to form flagella before hatching. They reassembled their flagella only after hatching from the sporangium suggesting that FAP265 plays an important role in flagellar reassembly after cell division.

## Introduction

Tubulin Polymerization Promoting Proteins (TPPPs) constitute a super family of proteins characterized by the presence of p25-alpha domain [1]. TPPP/p25 is the first member of this family of proteins identified earlier as a brain specific protein expressing in the oligodendrocytes [2, 3]. It is considered as a prototype of neomorphic moon lighting proteins wherein the same protein performs different functions under physiological and pathological conditions depending on the cellular location, concentration and interaction partners [4, 5]. TPPP is an unstructured basic protein which under physiological conditions promotes tubulin polymerization into normal and double-walled microtubules and induces microtubule bundling [6]. It modulates microtubule dynamics thereby inhibiting the assembly of mitotic spindle apparatus and affects cell proliferation [7, 8]. TPPP plays an important role in oligodendrocyte differentiation [9]. In non physiological conditions, TPPP is associated with diseases of central nervous system like Parkinson’s disease, multiple sclerosis and multiple system atrophy [10]. Enrichment of TPPP/p25 in inclusion bodies of synucleinopathies and its interaction with α-synuclein and β-amyloid, promoting their aggregation *in-vitro*, implicates this protein in the etiology of several neurological diseases [11, 12].

In humans, three paralogs of TPPP family of proteins were identified—TPPP1 (p25), TPPP2 (p18), TPPP3 (p20) [1]. Previous reports suggested that TPPP1 is involved in the differentiation of oligodendrocytes in the brain and TPPP3 was found to be a molecular marker of developing tendon sheath and synovial joints but the physiological role of TPPP2 is still unknown [9, 13] Microtubule bundling activity was associated with TPPP1 and TPPP3 but not with TPPP2 [14]. Further depletion of TPPP3 in HeLa cells resulted in multiple abnormalities during mitosis leading to cell death and depletion of TPPP3 in Lewis Lung Carcinoma cells inhibited tumour growth and metastasis [15, 16]. These results together suggest that paralogs of TPPP family proteins play an important role in development and disease.

Homologs of TPPP family of proteins were present in eukaryotic unicellular organisms to vertebrates but not in prokaryotes, fungi and plants [17]. Importantly TPPP homologs were found in the ciliary proteomes from various organisms including humans and *Chlamydomonas* and earlier report from Orosz and Ovadi using computational gene search and evidences from literature predicted that orthologs of TPPP family could be ciliary proteins [17, 18]. For example, TPPP1 was identified in the proteomic analysis of mouse photoreceptor sensory cilium [19]. TPPP3 was found in the proteome of motile cilia of bronchial epithelial cells [20]. Further TPPP3 was highly enriched (~74 fold) in the large scale transcriptomic analysis of human airway epithelial cells during mucociliary differentiation [21]. Apart from its presence in the ciliary proteomes, TPPP1 was also found to be down regulated, along with several ciliary genes, in gene expression studies carried out in primary ciliary dyskinesia patients [22]. These studies together suggest that TPPP family proteins are present in primary as well as motile cilia in various organisms but their role in the cilium remain unexplored.

In this study, we showed that *Chlamydomonas* homologue of TPPP, Flagellar Associated Protein (FAP265), localizes in the cytosol, basal bodies and flagella of vegetative *Chlamydomonas* cells. In dividing cells, FAP265 was present in the nucleus, and at cleavage furrow between two dividing cells. Further, null mutants of *Chlamydomonas* FAP265, *fap265*, showed severe defects in hatching. Daughter cells present within the mother sporangium failed to form flagella before hatching. Finally, they reassemble their flagella only after hatching from the sporangium suggesting that FAP265 plays an important role in flagellar reassembly after cell division.

## Results and discussion

### *Chlamydomonas* Flagellar Associated Protein, FAP265 is a TPPP family protein

*Chlamydomonas* homologue of TPPP family of proteins is a Flagellar Associated Protein, FAP265 which is a conserved hypothetical protein containing p25-alpha domain. FAP265 shows 35–37% sequence identity with human TPPP family proteins TPPP1, TPPP2 and TPPP3 (S1A and S1B Fig). In *Chlamydomonas* flagellar proteome, peptides for FAP265 were exclusively found in the membrane and matrix (M+M) fractions but not in axoneme, KCl and tergitol fractions indicating that the protein might bind loosely to flagellar microtubules [23]. Further, FAP265 was identified as a component of flagellar-basal body proteome through comparative proteomics approach [24]. Though the presence of FAP265 in the ciliary compartment was predicted earlier [17], neither its localization in *Chlamydomonas* flagellum nor its role in flagellar assembly or disassembly were explored.

### FAP265 localizes at the basal bodies and in the flagellum in vegetative *Chlamydomonas* cells

**Fig 1:**
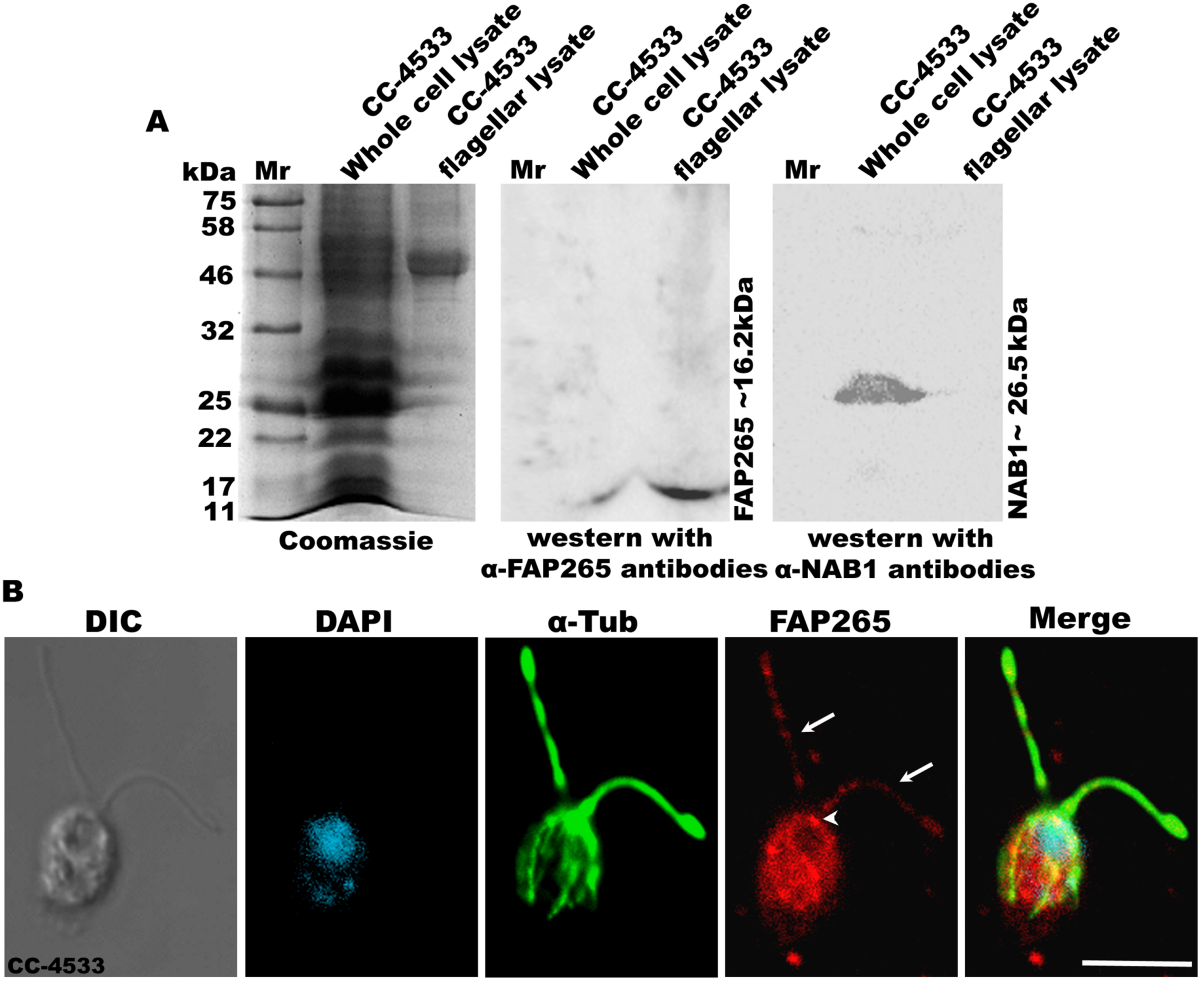
FAP265 localizes in flagella, basal bodies and cytoplasm in vegetative *Chlamydomonas* cells. (A) Coomassie gel showing lysates from whole cell and flagella of CC-4533 cells, separated on 12% SDS-PAGE. Western blotting using antibodies generated against recombinant FAP265 detects a specific band at ~16.2 kDa both in whole cell and flagellar fractions. Further, the blots were also probed with antibodies against NAB1 (Cytosolic marker). (B) Vegetative cells of wild type CC-4533 stained with antibodies against α-tubulin (green) and FAP265 (red). Arrows mark flagella and arrow head marks basal bodies. Nuclei were stained with DAPI (cyan). Scale: 10μm.

In order to determine the cellular localization of FAP265, we expressed recombinant FAP265 in *E*.*coli* BL21λ (DE3) and purified recombinant protein was used to raise polyclonal antibodies in rabbits. Western blotting showed that purified antibodies against FAP265 specifically detected a single band of ~16.2 kDa in whole cell and flagellar lysates suggesting the endogenous expression and presence of FAP265 in the flagellar compartment of *Chlamydomonas* cells (Fig 1A). The purity of the flagellar fractions was ascertained by microscopy as well as by western blotting using antibodies against Nucleic Acid Binding protein-1, NAB1 (cytoplasmic marker) (Fig 1A and S2 Fig). Further, immunofluorescence microscopy (IFM) studies showed the presence of FAP265 in the cytosol, at the basal bodies and in the flagella of vegetative *Chlamydomonas* CC-4533 cells (Fig 1B). FAP265 localizes in a punctate pattern along the length of the flagellum and the protein was excluded from the nucleus in steady state *Chlamydomonas* cells (Fig 1B).

### FAP265 localizes in the nucleus and cleavage furrow during cell division

**Fig 2:**
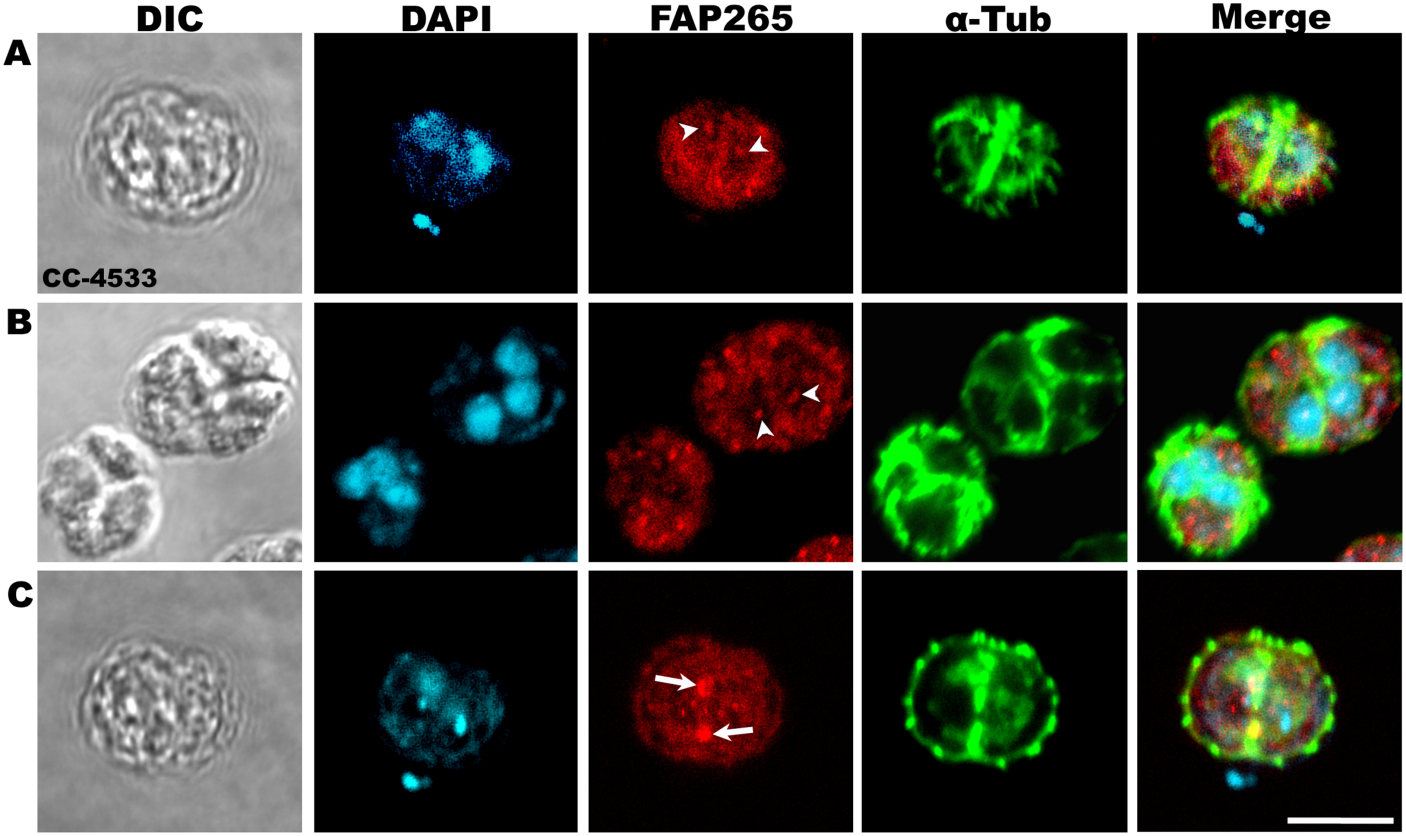
FAP265 localizes in the nucleus, (A, B) and at the cleavage furrow (C) in dividing CC-4533 cells. CC-4533 cells at different stages of cell division were fixed in methanol and stained with antibodies against α-tubulin (green) and FAP265 (red). Arrow heads mark nucleus and arrows mark cleavage furrow. Nuclei were stained with DAPI (cyan). Scale: 10μm.

In order to understand the role of FAP265, we studied intracellular localization of this protein during cell division in *Chlamydomonas*. In contrast to its exclusion from the nucleus in steady state vegetative cells, in dividing cells, FAP265 was found to be localized as a distinct spot within the nucleus (Fig 2A and 2B). Further, FAP265 was found to be present at the cleavage furrow region between the dividing daughter cells (Fig 2C). Earlier experiments using EGFP-TPPP/p25 in rat kidney cells have shown that mammalian TPPP localizes at the centrosomal region and spindle apparatus during metaphase and anaphase stages of cell division. During cytokinesis, TPPP was shown to be enriched at the cleavage furrow region [8]. Concomitant with their localization at the cleavage furrow, TPPP family proteins were also found to play an important role in cell division and cell survival [7, 8, 15, 16]. Hence the localization of FAP265 at the cleavage furrow in dividing *Chlamydomonas* cells, similar to mammalian TPPP family proteins, indicates that it may play an important role during cell division in *Chlamydomonas* cells.

### Loss of FAP265 results in failure of daughter cells to reassemble flagella after cell division

**Fig 3:**
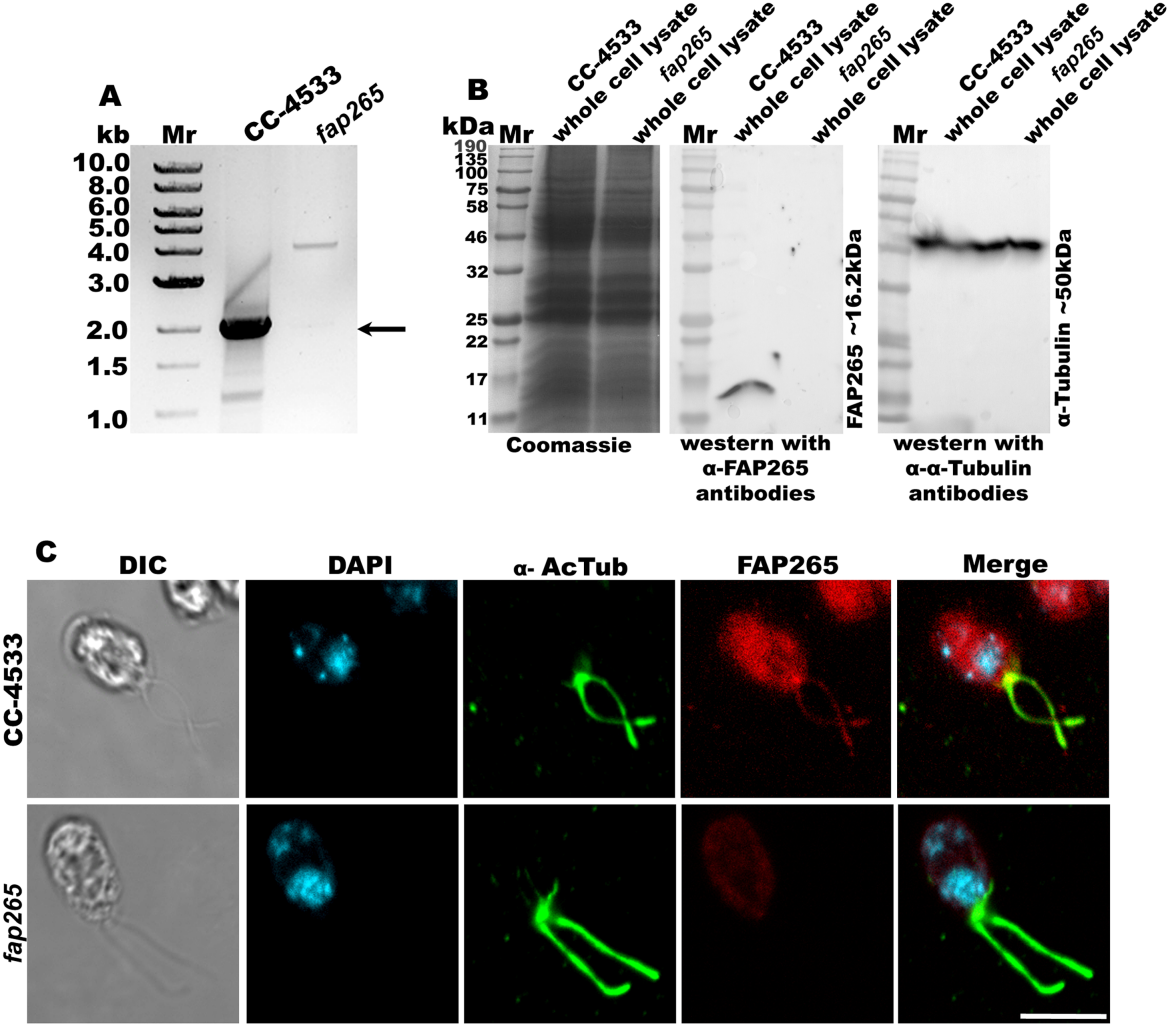
Validation of *fap265* mutant strain. (A) PCR amplification using FAP265 gene specific primers showed a specific band at ~2.0 kb with CC-4533 but not with *fap265* mutant genomic DNA. (B) Equal amount of whole cell lysates from CC-4533 and *fap265* mutant cells were separated on 12% SDS-PAGE; western blotting using antibodies against recombinant FAP265 detects a specific band at ~16.2 kDa in CC-4533 but not in *fap265* lysates. Western blotting using antibodies against α-tubulin was used as loading control. (C) CC-4533 and *fap265* cells were stained with antibodies against FAP265 (Red) and Acetylated-α-tubulin (ciliary marker, green). Nuclei were stained with DAPI (cyan). Scale: 10μm.

To test the cellular function of FAP265 protein, we obtained a *Chlamydomonas* insertional mutant for FAP265 gene, *fap265* (LMJ.RY0402.104619) from University of Minnesota [25]. The site of integration in *fap265* mutant in the insertional library was validated earlier by LEAP-Seq method and the confidence of insertion was determined to be 95% [25]. Further, validation of the *fap265* mutant strain was also carried out by us through PCR using gene specific primers against FAP265 gene and by western blotting. A specific 2.0 kb fragment was seen by PCR using genomic DNA isolated from wild type *Chlamydomonas* cells (CC-4533) which was absent in the mutant strain (Fig 3A). Further, western blotting using whole cell lysates of *fap265* mutant cells showed complete absence of protein compared with wild type cells (Fig 3B). Similarly, IFM analysis revealed that the protein is absent in mutant cells (Fig 3C) compared to its localization at the basal bodies and flagellum in control cells as described in earlier section. Together these results clearly showed that FAP265 is completely absent in the mutant cells we obtained.

**Fig 4:**
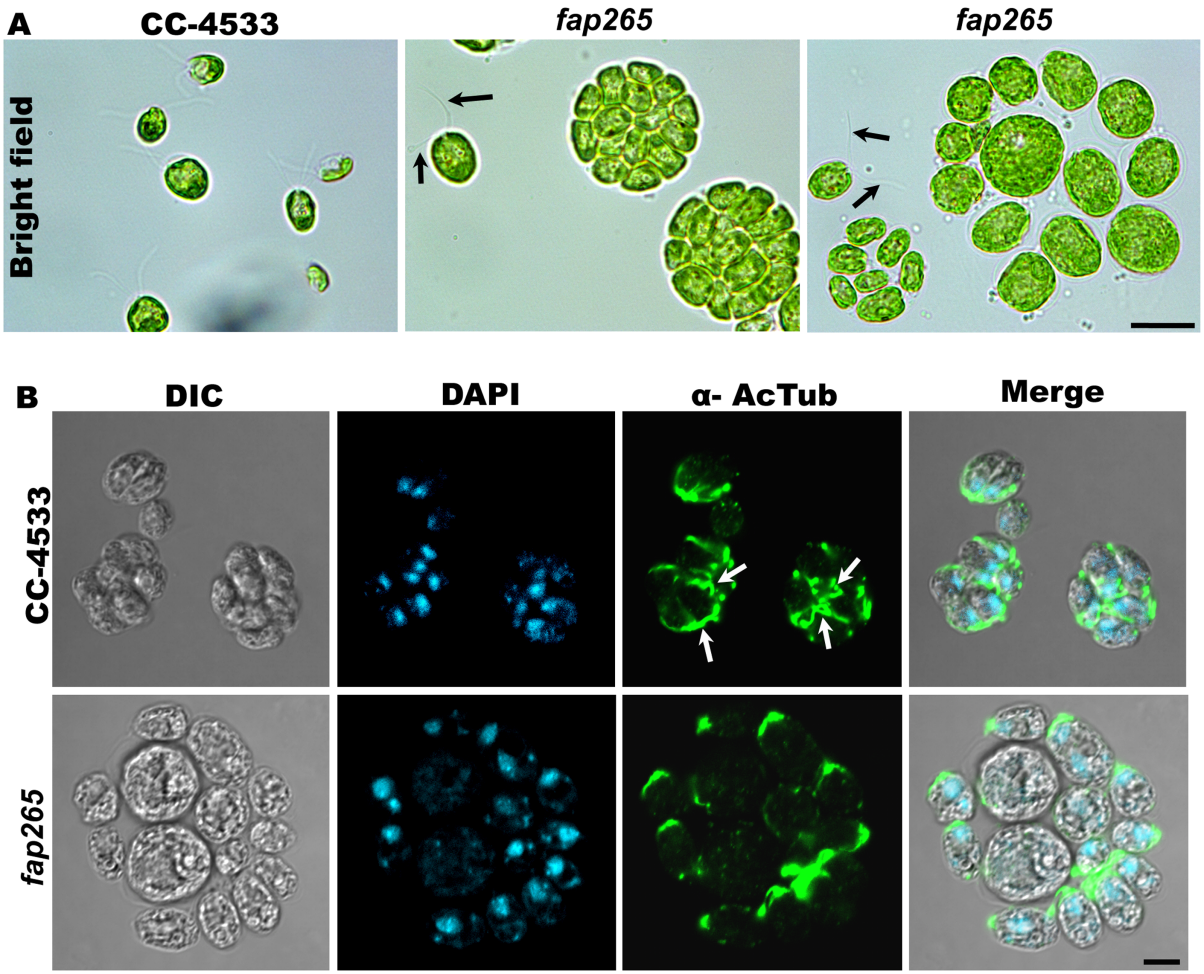
Loss of FAP265 results in severe hatching defects and failure of daughter cells to assemble flagella in sporangium. (A) Bright field micrographs showing sporangium containing large number of daughter cells at steady state in *fap265* mutant population compared with control cells. *fap265* mutant cells are significantly bigger compared with wild type cells. Arrows mark flagella. Scale: 20μm (B) *fap265* mutants and control cells in dividing stage were stained with antibodies against Acetylated-α-tubulin (ciliary marker, green). Nuclei were stained with DAPI. Scale: 10μm.

In order to assess the effect of gene deletion, we studied the growth characteristics of *fap265* mutant strain under standard culture conditions. The mutant cells showed severe hatching defect when cultured in TAP or minimal liquid culture media under constant shaking. Further, microscopic analysis of *fap265* mutant cells showed a population of large unhatched clumps containing ~16 daughter cells within each sporangium in contrast with the control strain wherein we found ~4–8 daughter cells within each sporangium (Fig 4A). In *fap265* mutants, the presence of mother sporangia can be observed throughout the growth phases but not in CC-4533 cells. Furthermore, mutant cells grow slower in liquid cultures when compared with control cells. While the turbidity (assessed by chlorophyll content) of CC-4533 culture increased steadily during the growth phase, the turbidity of *fap265* mutants remains very low from day 1–4 and increased rapidly later indicating that the mutant cells stay unhatched during the early stages of growth and hatched once they reach higher cell densities. Interestingly, the size of the *fap265* mutant cells was found to be significantly larger at steady state compared with control cells (Fig 4A). To ascertain this, anterio-posterior and lateral dimensions of the cells were measured from brightfield micrographs. In *fap265* mutants, both anterio-posterior (17.21±2.3μm; N = 52) and lateral lengths (13.68±2.8 μm; N = 52) were found to be significantly higher compared with CC-4533 cells which have anterio-posterior (11.43±1.32μm; N = 55) and lateral dimensions (8.76±1.28μm; N = 55). These results suggest that *fap265* mutant cells have phenotypic abnormalities and exhibit severe growth and hatching defects.

### Hatching defect in *fap265* mutants could be due to failure of daughter cells to reassemble flagella after cell division

Similar to mammalian cells, *Chlamydomonas* disassemble their flagella at G1 stage upon entry into cell cycle and flagellar reassembly occurs once the cell division is completed [26, 27]. IFM was carried out to understand the flagellar phenotype of the mutant cells. In *fap265* culture, daughter cells present within the mother sporangium failed to form flagella before hatching compared with the wild type cells where flagella can be clearly seen before hatching from the mother sporangium (Fig 4B). Further, daughter cells of *fap265* mutants reassembled flagella once released from the mother sporangium indicating that FAP265 might play a role in flagellar reassembly prior to hatching (Fig 4A). To confirm if FAP265 was involved in flagellar assembly, we tested flagellar regeneration in control cells and *fap265* mutants after deflagellation by pH shock. About 60% (63±2.64%; N = 300) of the mutant cells reassembled full length flagella (16.03±2.41 μm; N = 67) within 2 hours after deflagellation, compared to 95% (94.66±3.51%; N = 300) of control cells (15.60±2.11 μm; N = 67). These results together suggest that FAP265 might be involved in flagellar reassembly, after the cells complete the cell division, within the mother sporangium.

Earlier studies in *Chlamydomonas* showed that extra cellular bioactive vesicles are secreted from the flagellar membrane which contain vegetative lytic enzyme, sporangin, which is required for breaking the mother sporangium and hatching of daughter cells [28, 29]. Hence absence of flagella in the daughter cells of *fap265* mutants (when present within the mother sporangium) might result in reduced sporangin secretion which in turn could result in failure of daughter cells to hatch from the mother sporangium. Finally, the hatching of sporangia at later stages of growth in *fap265* cultures could be either due to the accumulation of large number of daughter cells inside each sporangium which could lead to mechanical disruption of sporangial wall or could be due to the trans-activity of sporangin carrying vesicles secreted by neighbouring cells or lysed mother sporangia. This also explains the rapid increase in cell density (turbidity) of mutant cells observed at late stages of growth in culture media.

TPPP family proteins function in stabilizing the microtubule network by their tubulin bundling activity. This is achieved by their direct interaction with microtubules [6]. Further TPPP1 was also found to inhibit Histone deacetylase, HDAC6 thereby promoting acetylation of microtubules [30]. Flagella are microtubule based structures and acetylation of conserved lysine (K40) residue on α-tubulin stabilizes the microtubules and is in turn important for ciliary assembly [31, 32, 33]. Furthermore the role of HDAC6 in flagellar assembly and disassembly is well established in several organisms [34, 35, 36]. Over expression of HDAC6 induces disassembly of primary cilia in Retinal Pigment Epithelial cells [36] and its inhibition in cholangiocarcinoma cell lines using HDAC6 inhibitors or shRNA restores ciliary assembly. Hence it can be envisaged that inhibition of HDAC6 by TPPP family proteins could play a positive role in ciliogenesis process.

TPPPs were classified into long type and short type based on the p25-alpha domain. While long type TPPPs contain the whole p25-alpha domain, the short type TPPPs possess a short p25-alpha domain which corresponds to the whole of the protein. Short type TPPPs also lack the Rossmann-like motif and most of the amino acids of the conserved microtubule-binding site which are present in long type TPPPs [14, 37]. Most of the vertebrate TPPPs studied earlier were long type TPPPs and *Chlamydomonas reinhardtii* FAP265 was reported to be a short type TPPP [37]. Hence our manuscript describes the first study showing the cellular role of a short type TPPP family protein.

In the present study, we showed the presence of a TPPP family protein, FAP265 in the cilium of eukaryotic green alga, *Chlamydomonas reinhardtii*. Redistribution of the protein during cell division and its role in flagellar reassembly prior to hatching from mother sporangium were discussed.

## Materials and methods

### Cultures

Wild type *Chlamydomonas reinhardtii* (CC-125mt+) was a kind gift from Prof. Joel Rosenbaum, Yale University, USA. *Chlamydomonas* insertional mutant for FAP265 gene (*fap265*) and corresponding control strain (CC-4533) were obtained from *Chlamydomonas* mutant library (Clip) [25]. CC-4533 was maintained on plates containing Tris-Acetate Phosphate (TAP) medium supplemented with 1.5% Agar and mutant strain was maintained on TAP-agar plate containing 20 μg/ml paramomycin. For experimentation, cultures were grown in liquid TAP medium [38] at 23°C with 12 hours light and dark cycles and constant shaking.

### Sequence analysis of *Chlamydomonas* FAP265

Identification of *Chlamydomonas* homologue of TPPP family proteins, FAP265 was done through BLASTp tool from NCBI database. Determination of conserved domains present in FAP265 was done through conserved domain database. Database of ciliary proteomes from various eukaryotes, Cildb V-3.0 [18], was used for identification of orthologs of TPPPs and predicting their ciliary localization.

### Purification of recombinant FA265 protein

For cloning of full length FAP265 gene, total cellular RNA was isolated from *Chlamydomonas reinhardtii* CC-125mt+ strain [39] and cDNA was synthesized from isolated mRNA using New England Biolabs One Strand Synthesis kit (Cat. No. E6300) as per manufacturer’s instructions. cDNA of ~498bp without a stop codon was amplified using forward (5’-GGATCCCATATGTCTGACGCTCTGAAGAATGCC-3’) and reverse (5’-AAGCTTGAATTTAACACCGCGCACGTCCG-3’) primers. The amplicon was cloned into cloning vector, pTZ57R/T (Thermo Scientific) and then subcloned at NdeI and HindIII sites of bacterial expression vector pET21A (Novagen). The cloned fragment was expressed in *E*.*coli* BL21λ (DE3) with a 6x-Histidine tag at the C-terminal end of the protein. Purification of the recombinant protein was carried out by Ni-NTA affinity purification as described in [40] with minor modifications. Elute fractions from the Ni-NTA resin were loaded on to 10% SDS-PAGE and bands representing FAP265 protein were excised from the gel, crushed in buffer containing (50mM Tris-Hcl, 150mM NaCl, 0.1mM EDTA, pH-7.5). FAP265 recombinant protein was eluted from the crushed gel by mechanical shaking overnight at 800 RPM on a vortex mixer (Eppendorf Thermomixer-C). Further, specificity of the recombinant FAP265 was determined by western blotting using antibodies against 6x-Histidine tag.

### Generation of polyclonal antiserum against recombinant FAP265 protein

Raising polyclonal antiserum against purified recombinant FAP265 protein was carried out as a paid service at Geniron Biolabs Pvt. Ltd. India. Animal Ethics Committee at Geniron Biolabs Pvt. Ltd. was approved by CPCSEA, Govt. of India (registration no. 1810/PO/RcBiBt/15/CPCSEA, 29.06.2015). A standard 75 days immunization protocol was used for raising polyclonal antisera. Briefly adult NewZealand White rabbits weighing 1.5–2.0 Kg, reared in house breeding facility of Geniron Biolabs Pvt. Ltd. were maintained under hygienic laboratory conditions, providing standard laboratory animal feed and water *ad libitum*. Rabbits were grouped and housed in stainless steel rabbit cages during the experiment. Further purified recombinant FAP265 antigen, thoroughly mixed with Freund’s complete adjuvant (for immunization) and Freund’s incomplete adjuvant (for boosters), was injected subcutaneously. For collecting preimmune and immune sera, blood was drawn from rabbits during the study period from central ear artery and marginal ear artery/vein using scalp after inducing anesthesia with ketamine (dose- 0.25 to 0.5 mg/kg) and Xyalazine (dose-0.1 to 0.2 ml) per rabbit. The drugs were administered 10–15 minutes prior to blood collection. Once the immunization protocol was completed, the animals were kept for rehabilitation for a period of one month and later used for other studies or breeding. Finally, anti-FAP265 antibodies were purified from animal bleeds by the blot-strip method using the purified recombinant FAP265 protein [41]. Sources and dilutions of antibodies used in the study were mentioned in supplementary S1 Table.

### Isolation of flagella from CC-4533 cells

Flagella were isolated from *Chlamydomonas* CC-4533 cells as described in [42] with minor changes. Cells grown in 2.4L liquid TAP media were harvested by centrifugation at 2500 rpm for 15 minutes at room temperature and the pellet was resuspended in 10mM HEPES, kept at 23°C for two hours with constant rocking. After pH shock, the cells were centrifuged at 2500 rpm for 10 minutes at 4°C and supernatant containing flagella was separated out. Using a serological pipette the flagellar suspension was under laid with HMDS containing 25% sucrose (10mM HEPES, 5mM MgSO_4_, 1mM DTT, 25% Sucrose, pH-7.4) and centrifuged at 2500 rpm for 10 minutes at 4°C. After centrifugation, the supernatant containing flagella was collected down to the sucrose interface and flagella were enriched by centrifuged at 14000 rpm for 20 minutes at 4°C. Finally, a white pellet of flagella was found at the bottom of the tube was resuspended gently in cold HMDEK buffer (10mM HEPES, 5mM MgSO_4_, 1mM DTT, 1mM EDTA, 25mM KCL, pH-7.2) containing protease inhibitors and stored at 80°C until use.

### Isolation of genomic DNA and PCR amplification to validate mutant

Genomic DNA from *Chlamydomonas* CC-4533 and mutant *fap265* cells was isolated as described in [43] and quantified using a nanodrop. Genomic DNA from CC-4533 and *fap265* was used for amplification of FAP265 gene using the following gene specific primers (Forward: 5’-GGATCCCATATGTCTGACGCTCTGAAGAATGCC-3’) (Reverse: 5’-AAGCTTGAATTTAACACCGCGCACGTCCG-3’). The PCR reaction was carried out in thermocycler from Biorad. Amplified products were analyzed by agarose gel electrophoresis. Images were captured on gel documentation system (Biorad).

### Western blotting

For western blotting, lysates of whole cells of CC-4533 and *fap265* were prepared following [44]. Briefly, fully grown cultures were pelleted at 3500 rpm for 5 minutes at room temperature. The pellet was washed with 1xPBS (Phosphate Buffered Saline) once and resuspended in sterile water. 0.1M DTT and 5% SDS were added and vortexed thoroughly. The suspension was centrifuged at 10000 rpm for 5 minutes at 25°C and supernatant was collected. Protein quantity was measured by Bradford assay (Biorad). Lysates from the cell pellets or isolated flagella were prepared by resuspending and boiling the supernatant collected from above for 5 minutes in 1xSDS sample buffer with intermittent vortexing. Samples were resolved on 12%SDS-PAGE and electroblotted onto nitrocellulose membrane. Antibodies against FAP265 were used for detecting endogenous FAP265. Mouse antibodies against α-tubulin were used as loading control. Antibodies against Nucleic Acid Binding protein-1 (NAB1) were used to check the purity of flagellar preparation. Source and dilutions of antibodies used in this study were mentioned in supplementary S1 Table. Finally, blots were developed by using chemiluminescent method. Images were captured on gel documentation system (Biorad).

### Immunofluorescence and brightfield microscopy

*Chlamydomonas* cells were processed for IFM as described in [45]. Briefly, cells were washed with 1x PBS twice and fixed in 90% methanol at -20°C for 20 minutes. Fixed cells were centrifuged and washed twice with 1x PBS and adhered on poly-L-Lysine coated cover slips. After 15 minutes, excess cell suspension was removed and the coverslips were dipped in methanol for 5 minutes and air dried. Upon rehydration, the cells were blocked with blocking buffer (0.5% BSA in 1xPBS with 5% normal goat serum) and incubated with primary antibodies as per dilutions mentioned in supplementary S1 Table. Cover slips were finally mounted using prolong diamond anti-fade mounting media containing DAPI (Invitrogen; P36966) and images were captured on laser scanning confocal microscope (NIKON) using a 100x1.4NA (oil) plan apochromate lens. For brightfield microscopy, cells were fixed in 2% paraformaldehyde and images were captured on light microscope (NIKON Eclipse E200) using 100x1.2NA (oil) plan lens. Cell sizes, in terms of anterio-posterior and lateral dimensions, were measured from the captured images using NIS elements software.

## Supporting information

S1 Fig Identification of *Chlamydomonas* homologue of human TPPPs. (A) Multiple sequence alignment between TPPP1/P25, TPPP2/P18, TPPP3/P20 and *Chlamydomonas* FAP265 using Clustal Omega [1]. (B) Domain architecture of FAP265 predicted through Conserved Domain Database.
(DOCX)

S2 Fig Purity of the flagellar preparation. Phase contrast image showing the presence of flagella but not cell bodies in the flagellar preparation used for western blotting.
(DOC)

S1 Table Table showing the list of antibodies used in the study. (DOC)
