# Supplementary material for: Chlamydomonas FAP265 is a tubulin polymerization promoting protein, essential for flagellar reassembly and hatching of daughter cells from the sporangium
Source: PLoS One. 2017 Sep 20;12(9):e0185108. doi: 10.1371/journal.pone.0185108 (PMC5607191; doi:10.1371/journal.pone.0185108)
Supplement: S1 Fig — (A) Multiple sequence alignment between TPPP1/P25, TPPP2/P18, TPPP3/P20 and Chlamydomonas FAP265 using Clustal Omega [1]. (B) Domain architecture of FAP265 predicted through Conserved Domain Database. (DOCX) [file pone.0185108.s001.docx]

**S1Fig.** **Identification of *Chlamydomonas* homologue of human TPPPs.** (A) Multiple sequence alignment between TPPP1/P25, TPPP2/P18, TPPP3/P20 and *Chlamydomonas* FAP265 using Clustal Omega [1]. (B) Domain architecture of FAP265 predicted through Conserved Domain Database.

**S1A Fig. Multiple Sequence Alignment between human TPPPs and *Chlamydomonas* FAP265 using Clustal Omega.**

TPPP2 ----------------------------------------------MASEAEKTFHRFAA

TPPP1 MADKAKPAKAANRTPPKSPGDPSKDRAAKRLSLESEGAGEGAAASPELSALEEAFRRFAV

TPPP3 -----------------------------------------MAASTDMAGLEESFRKFAI

FAP265 ----------------------------------------------MSDALKNAFIAFAS

:::* **

TPPP2 FGESSSSGTEMNNKNFSKLCKDCGIMDGKTVTSTDVDIVFSKVKAKNARTITFQQFKEAV

TPPP1 HGDARATGREMHGKNWSKLCKDCQVIDGRNVTVTDVDIVFSKIKGKSCRTITFEQFQEAL

TPPP3 HGDPKASGQEMNGKNWAKLCKDCKVADGKSVTGTDVDIVFSKVKGKSARVINYEEFKKAL

FAP265 YGKGQMMKQDMDNKNFSKCIKDSGILD-KVITSTEVDITFMKVKAKTDRTINFAQFCTAL

.*. :*..**::* **. : * : :* *:***.* *:*.*. *.*.: :* *:

TPPP2 KELGQKRFKGKSPDEVLENIYGLMEGKDPATTGATKATTVGAVDRLTDTSKYTGTHKERF

TPPP1 EELAKKRFKDKSSEEAVREVHRLIEGKAPIISGVTKAISSPTVSRLTDTTKFTGSHKERF

TPPP3 EELATKRFKGKSKEEAFDAICQLVAGKEPANVGVTKAKTGGAVDRLTDTSRYTGSHKERF

FAP265 EHFAAKRGVSV------DSLHAKVEAASPTS----NATQAEAVKFHDDKNLYTGVYKNGG

:.:. ** . : : . * :* :*. *.. :** :*:

TPPP2 DES---GKGKGIAGREEMTDNTGYVSGYKGSGTYDKKTK---

TPPP1 DPS---GKGKGKAGRVDLVDESGYVSGYKHAGTYDQKVQGGK

TPPP3 DES---GKGKGIAGRQDILDDSGYVSAYKNAGTYDAKVKK--

FAP265 PTNIDKQAAGGLAGHLDRSP--ADVRGVKF------------

. . * **: : . * . *

*Chlamydomonas* FAP265 shows 35-37% sequence identity and ~55% similarity with Human TPPP family proteins.

**S1B Fig.**

**Conserved architecture of *Chlamydomonas* FAP265:**

p25-alpha domain pfam05517

Interval 6-151

E-Value 4.68e-46


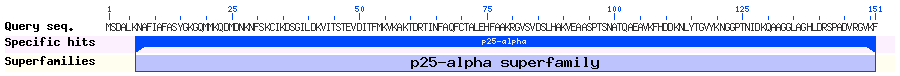


­


**References**

1. [Sievers, F](https://www.ncbi.nlm.nih.gov/pubmed/?term=Sievers%20F%5BAuthor%5D&cauthor=true&cauthor_uid=21988835)., [Wilm, A](https://www.ncbi.nlm.nih.gov/pubmed/?term=Wilm%20A%5BAuthor%5D&cauthor=true&cauthor_uid=21988835)., [Dineen, D](https://www.ncbi.nlm.nih.gov/pubmed/?term=Dineen%20D%5BAuthor%5D&cauthor=true&cauthor_uid=21988835)., [Gibson, T. J](https://www.ncbi.nlm.nih.gov/pubmed/?term=Gibson%20TJ%5BAuthor%5D&cauthor=true&cauthor_uid=21988835)., [Karplus, K](https://www.ncbi.nlm.nih.gov/pubmed/?term=Karplus%20K%5BAuthor%5D&cauthor=true&cauthor_uid=21988835)., [Li, W](https://www.ncbi.nlm.nih.gov/pubmed/?term=Li%20W%5BAuthor%5D&cauthor=true&cauthor_uid=21988835). et al Fast, scalable generation of high-quality protein multiple sequence alignments using Clustal Omega. [Mol Syst Biol.](https://www.ncbi.nlm.nih.gov/pubmed/21988835) 2004; 7:539
