# Supplementary material for: Chlamydomonas FAP265 is a tubulin polymerization promoting protein, essential for flagellar reassembly and hatching of daughter cells from the sporangium
Source: PLoS One. 2017 Sep 20;12(9):e0185108. doi: 10.1371/journal.pone.0185108 (PMC5607191; doi:10.1371/journal.pone.0185108)

**S2Fig. Purity of the flagellar preparation.** Phase contrast image showing the presence of flagella but not cell bodies in the flagellar preparation used for western blotting.


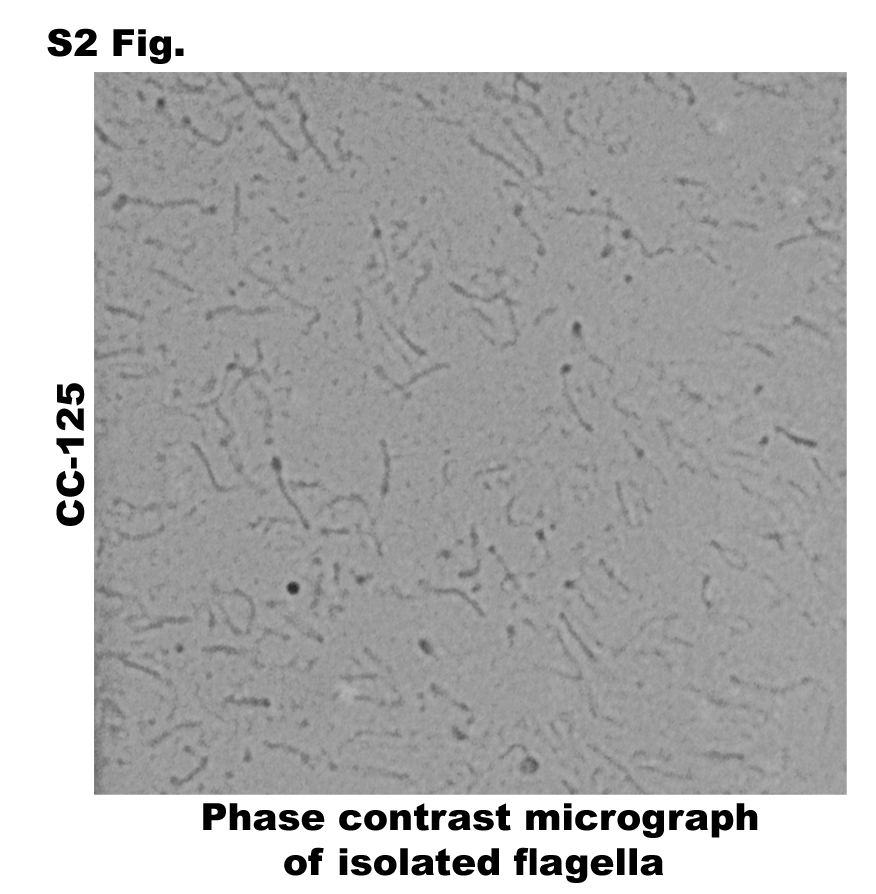

Supplement: S2 Fig — Phase contrast image showing the presence of flagella but not cell bodies in the flagellar preparation used for western blotting. (DOC) [file pone.0185108.s002.doc]
