# Supplementary material for: Chlamydomonas FAP265 is a tubulin polymerization promoting protein, essential for flagellar reassembly and hatching of daughter cells from the sporangium
Source: PLoS One. 2017 Sep 20;12(9):e0185108. doi: 10.1371/journal.pone.0185108 (PMC5607191; doi:10.1371/journal.pone.0185108)
Supplement: S1 Table — (DOC) [file pone.0185108.s003.doc]

**S1Table.** Table showing the list of antibodies used in the study.

# S1Table: List of antibodies used in the study

| **S. No** | **Antigen** | **Source** | **Dilution** |
| --- | --- | --- | --- |
| 1. | Mouse Acetylated-α-tubulin | Sigma (T7451) | 1:1000 (IF) |
| 2. | Mouse α-tubulin | Santacruz (SC-23948) | 1:500 (IF)  1:5000 (WB) |
| 3. | Rabbit CrFAP265 | Raised in rabbit | 1:100 (IF);  1:500 (WB) |
| 6. | Rabbit Nucleic acid binding protein 1(NAB1) | Agrisera (AS08333) | 1:10000 (WB) |
| 7. | Rabbit His probe | Santacruz (SC804) | 1:5000 (WB) |

IF – Immunofluorescence and WB- western blotting
